# Supplementary material for: Pseudomonas aeruginosa population dynamics in a vancomycin-induced murine model of gastrointestinal carriage
Source: mBio. 2025 Apr 10;16(5):e03136-24. doi: 10.1128/mbio.03136-24 (PMC12077156; doi:10.1128/mbio.03136-24)
Supplement: Supplemental material — Supplemental methods and figures. [file mbio.03136-24-s0001.docx]

**Supplemental Methods**

## Long-term carriage assessment

For long-term carriage experiments, six- to eight-week-old mice (male and female) received seven daily IP injections of vancomycin and were orally gavaged with 10^5.7+/-0.3^ CFU of strain PABL048 as described in the main Methods section. Starting the day after the exposure to bacteria, cages were changed daily for the first 14 days of the experiment, then at least 3 times per week thereafter. Assessment of GI carriage was performed as described in the main Methods section.

## Carriage assessment following metronidazole pre-treatment

Six- to eight-week-old female mice received 600 µL of metronidazole (275 mg/kg, Abcam, Waltham, MA) daily IP for seven days. The metronidazole dosage was allometrically scaled based on a total human daily dose of 22.5 mg/kg (1). On the fifth day of antibiotic treatment, mice were gavaged with 10^7.2^ CFU of strain PABL048 as described in the main Methods section. Starting the day after the exposure to bacteria, cages were changed daily, and assessment of GI carriage was performed as described in the main Methods section.

## Histopathology analysis

Six- to eight-week-old female mice were separated into 3 groups: one group received 7 daily IP vancomycin injections and an orogastric gavage with strain PABL048 as described in the main Methods section, one group received 7 daily IP vancomycin injections and a mock orogastric gavage with 50 µL PBS, and one group received 7 daily IP PBS (200 µL) injections and an orogastric gavage with PABL048. Orogastric gavage with PABL048 was performed using 10^7.1^ CFU in 50 µL. On day 3 post-gavage, feces were collected to assess the extent of carriage. Mice were sacrificed, and organs from the GI tract were harvested and fixed in 10% formaldehyde for 48 hours. Samples were embedded in paraffin, sectioned into 4 µm-thick slices, and stained with hematoxylin and eosin by the Mouse Histology and Phenotyping Laboratory of Northwestern University. Samples were imaged using an Olympus BX45 microscope with an Olympus DP28 digital camera. Pictures were visualized using Olympus cellSens imaging software (version 4.2) and analyzed by a pathologist for the presence of inflammatory cells and evidence of tissue damage.

## Bacterial dissemination assessment

Six- to eight-week-old female mice received 7 daily IP injections of vancomycin and were orally gavaged with 10^7.2+/-0.2^ CFU of strain PABL048 as described in the main Methods section. Mice were sacrificed on day 3, 7, or 14 post-gavage, and the following organs were collected: stomach, small intestine, cecum, colon, feces, gallbladder, liver, spleen, and lungs. Dissection tools used to collect organs from the GI tract were kept separately from tools used for other tissues. Organs were homogenized in 1 mL of PBS using a bead blaster, centrifuged for 30 sec at 1,100 x *g*, and the supernatant was serially diluted and plated on VBM agar for CFU enumeration.

## Construction of the barcoded library

The construction of the barcoded library of strain PABL012 was previously described by Bachta *et al.* (2). Briefly, a pminiCTX_STAMP_ plasmid was created from a pminiCTX1 backbone (3) to contain a gentamicin resistance cassette for antibiotic selection. Collections of randomly generated 30 bp barcodes were inserted into this plasmid, and the plasmid pool was transformed into *E. coli* SM10 λpir, which was used to perform conjugation with PABL012. This resulted in the integration of the pminiCTX_STAMP_ plasmid at the *attB* site of *P. aeruginosa,* hence creating the PABL012_pool_. The growth of the barcoded PABL012 library, the stability of the barcode insertions and the calibration curve for the STAMP study have been previously published (2).

## STAMPR analysis

Bacteria recovered on the 150-mm-diameter VBM plates were processed as previously described (2). Briefly, CFU were scraped off the plates and resuspended in PBS, genomic DNA was harvested using a cell DNA purification kit and a Maxwell^TM^ 16 Instrument (Promega, Madison, WI), and the concentration was adjusted to 10 ng/µL. The barcodes were PCR amplified using primer P47 with primer P48 or P51-P73 (Supplementary Table 2). Amplicons were electrophoresed through a 1.5% agarose gel, extracted from the gel (QIAquick® Gel Extraction Kit, Qiagen, Germantown, MD), quantified (high sensitivity Quant-IT dsDNA Assay kit, Invitrogen, Carlsbad, CA), and pooled at equimolar concentrations (4 nM final concentration). The pooled samples were sequenced on a MiSeq instrument (50-cycle Miseq Reagent kit v2, Illumina, San Diego, CA) following the manufacturer’s instructions (4) and using primer P49 (Supplementary Table 2) as a custom primer.

Sequencing reads (.fastq files) were trimmed using CLC Genomics Workbench and mapped to a reference list of barcodes using default parameters. Read counts per barcode were exported as .csv files and imported into the STAMPR pipeline in R. The founding population size was estimated by calculating N_s_ using the STAMPR analysis pipeline as previously described (5, 6). Briefly, barcode read counts were first corrected for index hopping by subtracting 0.05% of reads derived from barcodes in other samples in the sequencing run and then converted to frequencies. Frequency gaps higher than 10-fold and frequency breaks delineating sub-populations were identified as previously described (6), and barcode frequencies lower than the threshold were designated as noise. The input library was then resampled based on the sequencing depth of the output sample. This new resampled input library was further used to calculate N_s_, defined as the sampling depth (from resampling with a multinomial distribution) required to observe a specific number of unique barcodes in an output sample. The pipeline used to analyze this data set is available at: <https://github.com/hullahalli/stampr_rtisan>.

The genetic distance (GD) between 2 samples (A and B) was calculated using the Cavalli-Sforza chord distance (7). Lower GD values indicate increased barcode similarity between samples. The fractional resilient genetic distance (FRD) was calculated using: $\frac{ln({RD}_{A-B}+1)}{\ln(Number of barcodes in B+1)}$. RD_A-B_ is calculated by first ordering pairs of barcode frequency vectors by geometric means of each barcode and calculating genetic distance (iteration 0). Then, the most abundant shared barcode by geometric mean is removed, and GD is calculated again (iteration 1). These iterations are repeated for *n* number of times, where *n* is the smaller value of the number of unique barcodes in sample A or the number of unique barcodes in sample B. The number of iterations that yield a GD < 0.8 is defined as RD. The column names in the FRD heatmaps correspond to the organ of reference (B in the above formula) (Fig. 6E-G). High FRD values indicate that most bacterial clones are shared between samples.

**Recovery of *P. aeruginosa* from the GI tract at early times following inoculation** Six- to eight-week-old female mice received 5 daily IP injections of vancomycin and were orally gavaged with 10^6.1^ CFU of strain PABL012 as described in the main Methods section. Mice were sacrificed at 1 or 6 hours post-gavage, and the following organs were collected: stomach, small intestine, cecum, colon, feces. All feces produced by the mice were collected from the time of orogastric gavage to the termination of the experiment. Organs were homogenized in 1 mL of PBS using a bead blaster, centrifuged for 30 sec at 1,100 x *g*, and the supernatant was serially diluted and plated on VBM agar for CFU enumeration. To decrease the limit of detection, 250 µL of homogenized tissues were also spread on 150-mm-diameter VBM plates.

## Statistical analysis

Statistical analyses were performed using GraphPad Prism software (version 10.2.3). Details are provided in the figure legends.

**Supplemental Figures**


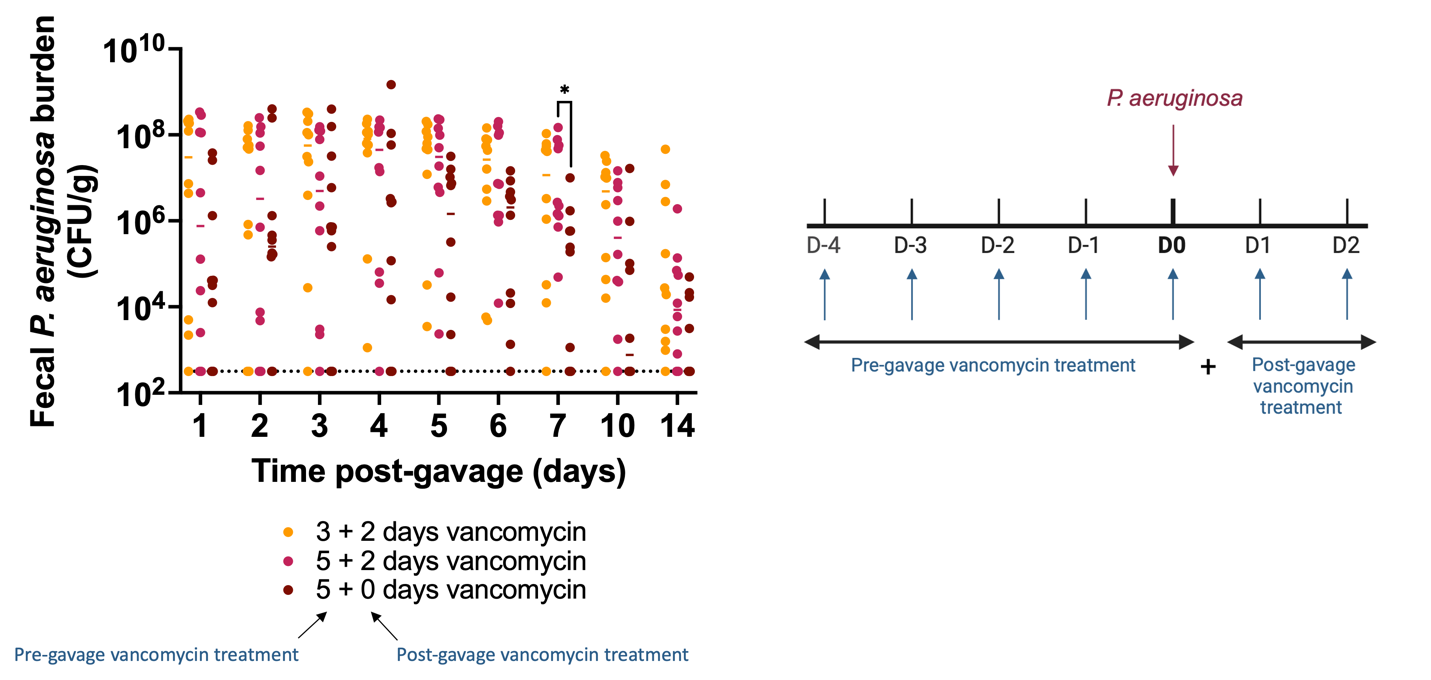


**Supplemental Figure 1: GI carriage of *P. aeruginosa* obtained with various regimens of vancomycin treatment*.*** Mice received daily injections of vancomycin for various times before and after orogastric gavage (“x + y days” with x = the number of days of vancomycin injections prior to and on the day of orogastric gavage*,* and y = the number of days of vancomycin injections after the bacterial inoculation). Orogastric gavage was performed with 10^5.8+/-0.2^ CFU of strain PABL048. Each symbol represents one mouse. Solid horizontal lines indicate medians. The experiment was performed twice (combined results shown; n = 10). The dotted line indicates the limit of detection. There were no significant differences between groups unless otherwise noted with *p ≤ 0.05 (Mann-Whitney tests with Bonferroni-Dunn correction). Significant differences were not detected for any of the time points between mice treated with 5 + 2 days and 3+ 2 days of vancomycin.


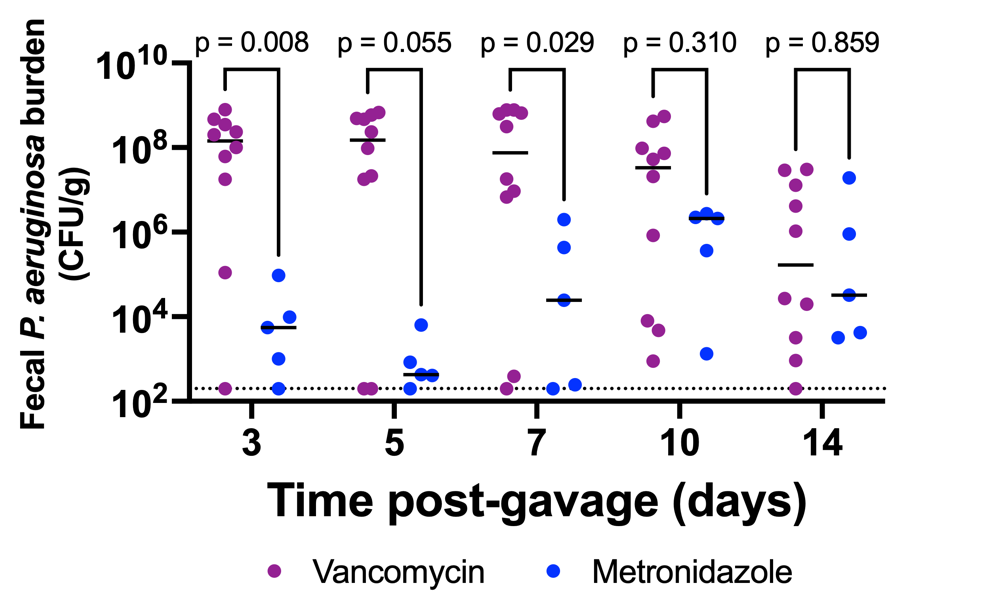


**Supplemental Figure 2: GI carriage of *P. aeruginosa* obtained with various regimens of antibiotic treatment.** Mice were treated with either vancomycin (purple) or metronidazole (blue) for 7 days. On the fifth day of treatment, mice received 10^7.2+/-0.05^ CFU of PABL048 through orogastric gavage. The experiment was performed twice and the results combined for the vancomycin-treated group (n = 10 animals/group), and once for the metronidazole-treated group (n = 5 animals/group). Each symbol represents one mouse. Lines indicate medians. The dotted line indicates the limit of detection. p-values are indicated (two-tailed Mann-Whitney test).


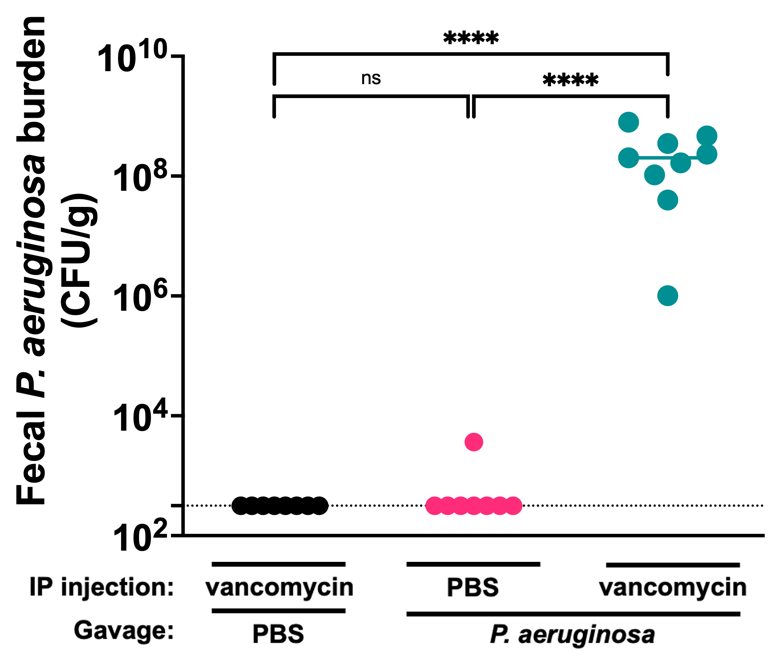


**Supplemental Figure 3: Fecal burden of strain PABL048 at day 3 post-inoculation.** Mice were treated with either PBS (pink) or vancomycin (black and teal) for 7 days. On the fifth day of treatment, mice received either PBS (black) or 10^7.1+/-0.1^ CFU of PABL048 through orogastric gavage (pink and teal). The experiment was performed twice and the results combined (n = 8-9 animals/group). Each symbol represents one mouse. Lines indicate medians. The dotted line indicates the limit of detection. p-values were calculated using two-tailed Mann-Whitney tests: ns = non-significant, ****p < 0.0001.


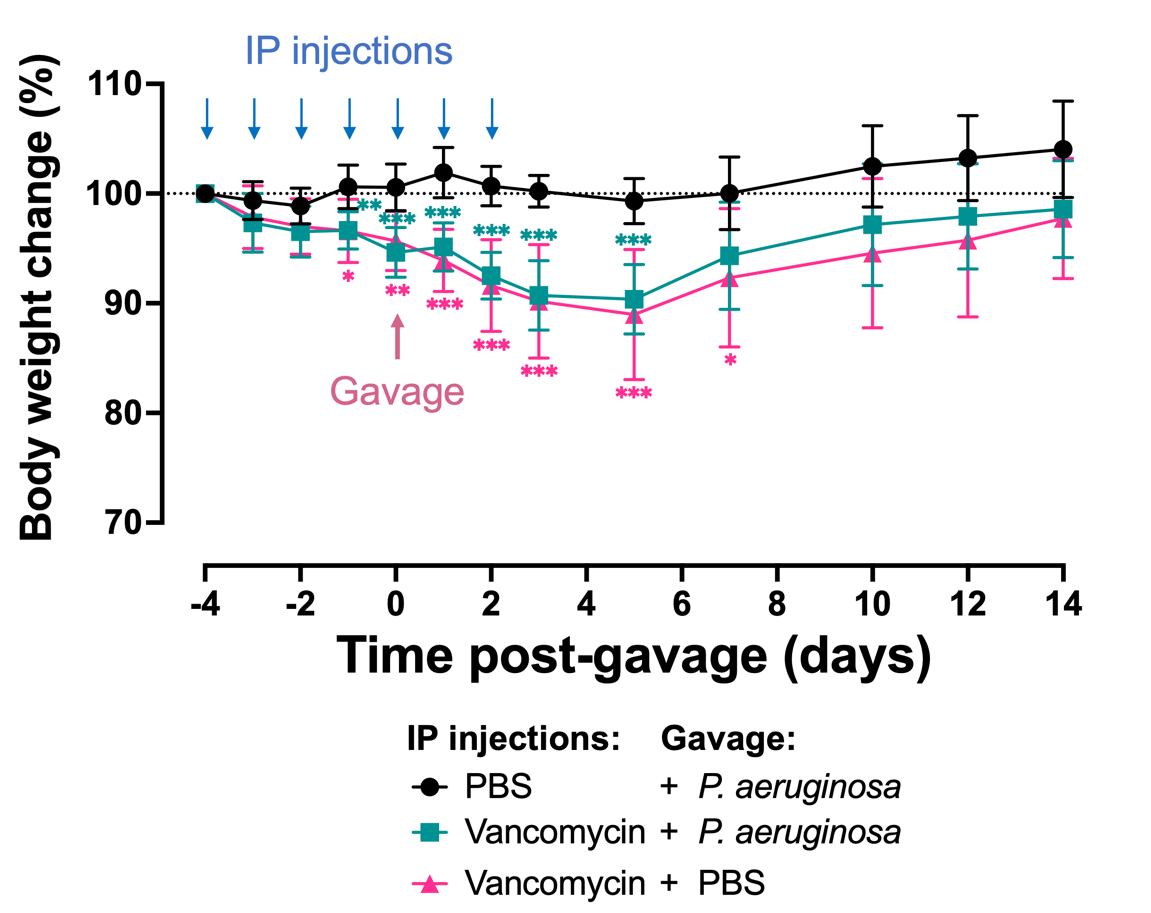


**Supplemental Figure 4: Body weights of mice following administration of vancomycin and/or *P. aeruginosa*.** Mice were treated with either PBS (pink) or vancomycin (black and teal) for 7 days. On the fifth day of treatment (day 0), mice received either PBS (black) or 10^7.2^ CFU of PABL048 via orogastric gavage (pink and teal). Weight changes were calculated with reference to day -4 (set at 100%). Dotted line indicates starting weight point. The experiment was performed twice and the results combined (n = 10 animals/group). Statistical analysis was performed for each time point by comparing vancomycin-treated groups to the PBS-treated control group (unpaired t-tests with Bonferroni-Dunn correction for multiple comparisons). Unless indicated, no significant differences were observed between groups. *adjusted p ≤ 0.05, **adjusted p ≤ 0.01, ***adjusted p ≤ 0.001.


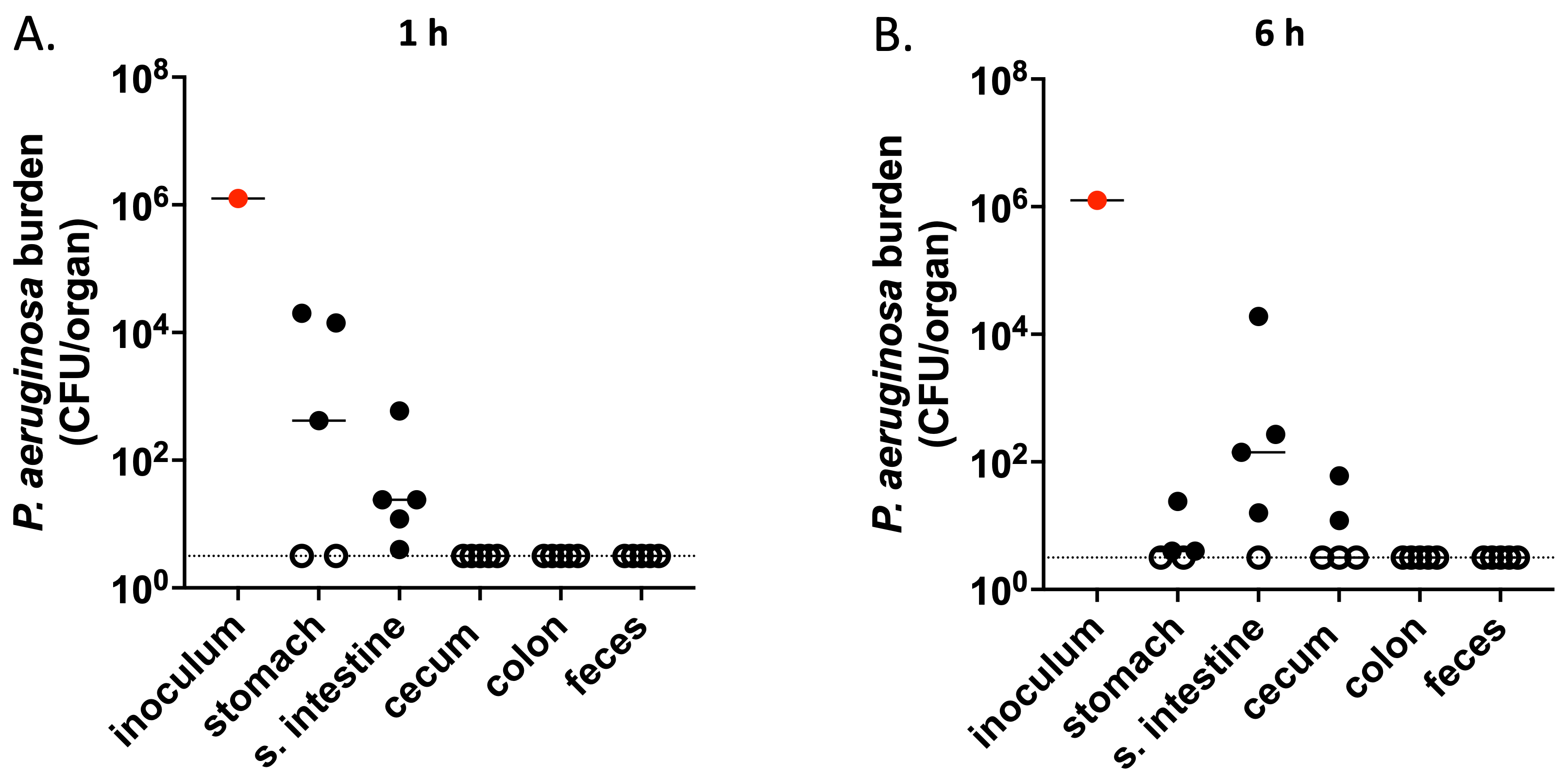


**Supplemental Figure 5: Recovery of *P. aeruginosa* from the GI tract at early times following inoculation**. *P. aeruginosa* burden in GI tissues of mice gavaged with PABL012. Mice were sacrificed at (A) 1 h (n = 5) or (B) 6 h (n = 5) post-orogastric gavage with 10^6.1^ CFU of PABL012, and bacterial CFU in the organs were enumerated by plating. The experiment was performed once. Red circles represent the inoculums. Each black circle represents one mouse. Solid horizontal lines indicate medians. The horizontal dotted line indicates the limit of detection. Open circles represent tissues with CFU below the limit of detection.


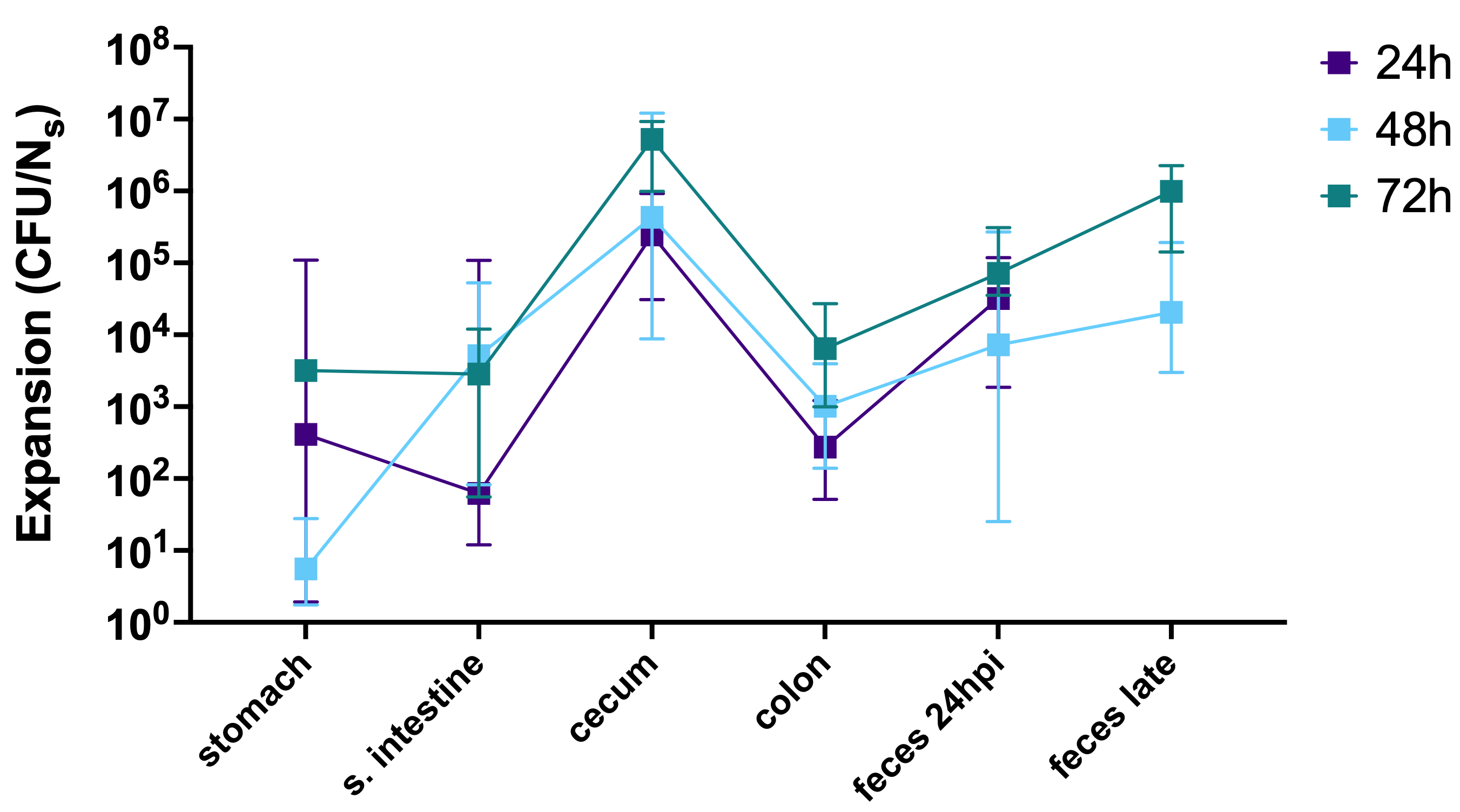


**Supplemental Figure 6: Ratio of bacterial recovery vs. founding population in GI sites.** Tissues were harvested at 24 (purple, n = 5), 48 (blue, n = 4) or 72 hours (green, n = 3) after orogastric gavage with PABL012_pool_. Fecal samples were collected at 24 hpi (“feces 24 hpi”) regardless of the ending timepoint. Additional terminal fecal sample timepoints were available for animals that had organs harvested at 48 or 72 hpi (“feces late”). CFU/N_s_ ratios were calculated. Squares represent medians, and error bars represent the 95% confidence intervals. There were no significant differences between the timepoints (paired t-tests).


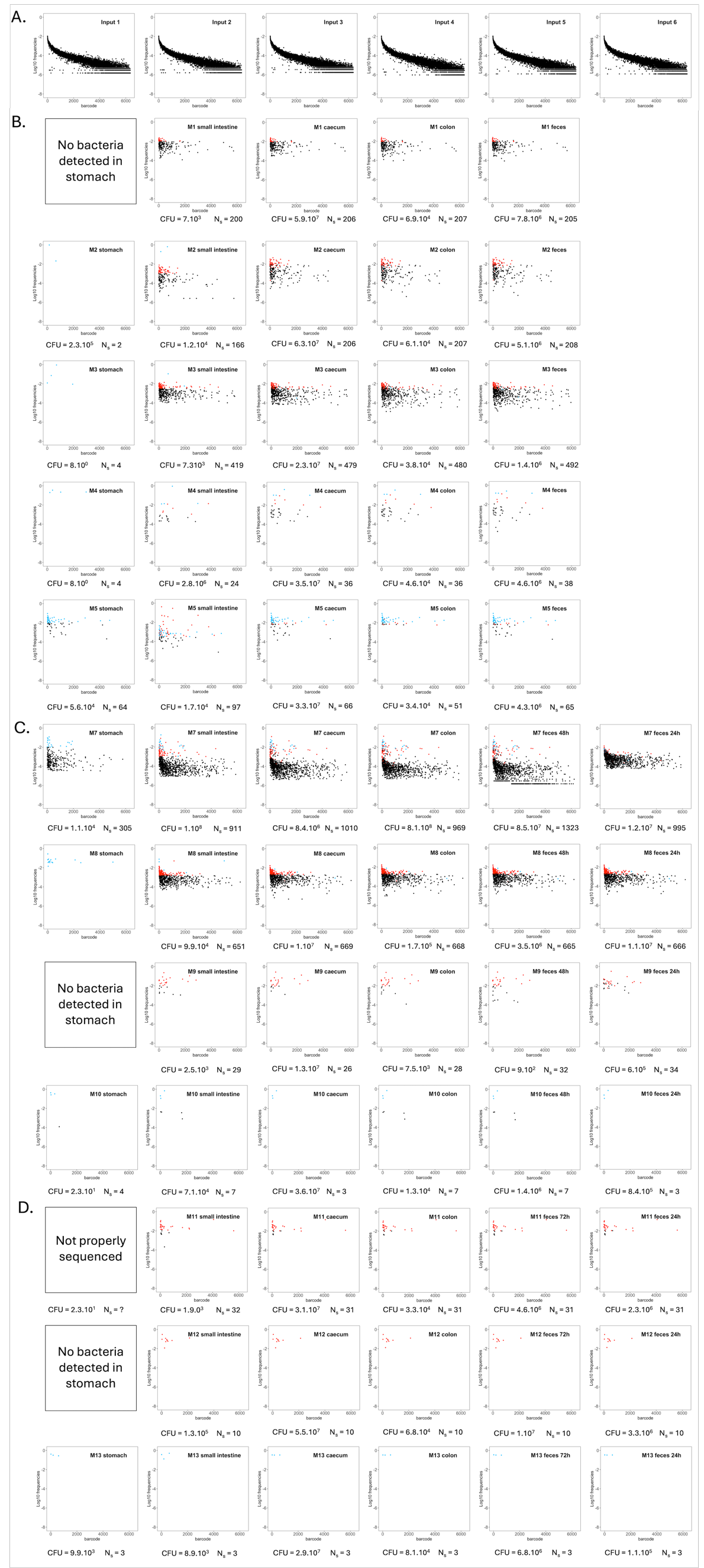

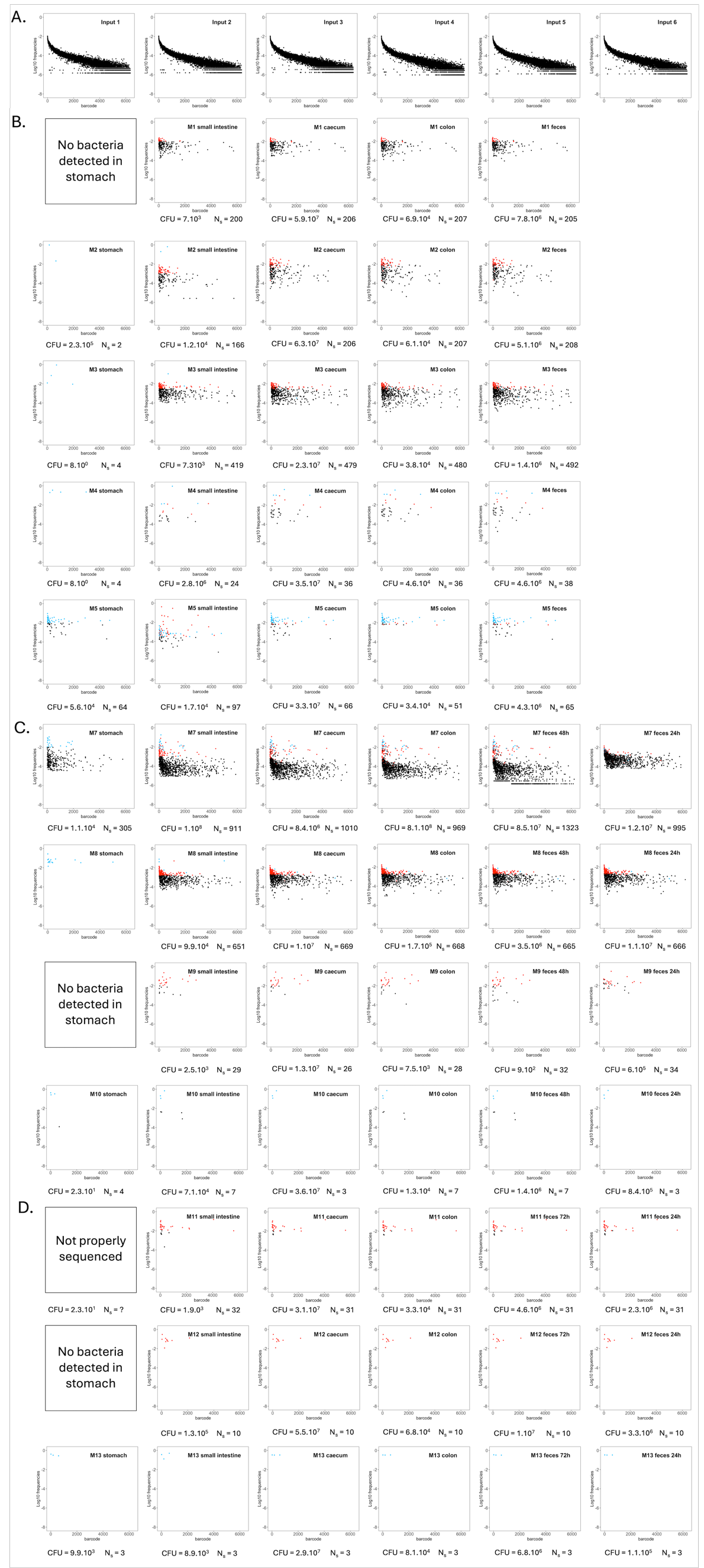


**Supplemental Figure 7: Barcode frequency distributions of *P. aeruginosa* bacteria recovered from mice following orogastric inoculation.** The frequencies of unique barcodes in each bacterial population from different sites are shown. (A) Inoculum samples. Barcode frequency was analyzed in 26 aliquots taken from the STAMP library, which were used to assess the composition of the inoculum. Six representative frequency distributions are shown. (B-D) Barcode frequency distributions after noise removal for the output samples from mice sacrificed at (B) 24, (C) 48 or (D) 72 hours post-orogastric gavage. Each dot represents the frequency at which one specific barcode was detected. For each mouse (“M#”), dots representing the most frequent clones identified in the stomach are colored blue in all organs, and dots representing the most frequent clones identified in the small intestine are colored red.

# REFERENCES

1. U.S. Department of Health and Human Services. 2005. Guidance for Industry - Estimating the Maximum Safe Starting Dose in Initial Clinical Trials for Therapeutics in Adult Healthy Volunteers. Food and Drug Administration.

2. Bachta KER, Allen JP, Cheung BH, Chiu C-H, Hauser AR. 2020. Systemic infection facilitates transmission of *Pseudomonas aeruginosa* in mice. 1. Nat Commun 11:543.

3. Hoang TT, Kutchma AJ, Becher A, Schweizer HP. 2000. Integration-Proficient Plasmids for *Pseudomonas aeruginosa*: Site-Specific Integration and Use for Engineering of Reporter and Expression Strains. Plasmid 43:59–72.

4. Illumina. MiSeq System Denature and Dilute Libraries Guide (15039740).

5. Fakoya B, Hullahalli K, Rubin DHF, Leitner DR, Chilengi R, Sack DA, Waldor MK. Nontoxigenic *Vibrio cholerae* Challenge Strains for Evaluating Vaccine Efficacy and Inferring Mechanisms of Protection. mBio 13:e00539-22.

6. Hullahalli K, Pritchard JR, Waldor MK. 2021. Refined Quantification of Infection Bottlenecks and Pathogen Dissemination with STAMPR. mSystems 6:e00887-21.

7. Cavalli-Sforza LL, Edwards AWF. 1967. Phylogenetic analysis. Models and estimation procedures. Am J Hum Genet 19:233–257.
