# Supplementary material for: Pseudomonas aeruginosa population dynamics in a vancomycin-induced murine model of gastrointestinal carriage
Source: mBio. 2025 Apr 10;16(5):e03136-24. doi: 10.1128/mbio.03136-24 (PMC12077156; doi:10.1128/mbio.03136-24)
Supplement: Table S1 — Strains used in this study. [file mbio.03136-24-s0002.pdf]

Supplemental Table 1. Strains used in this study

Species	Strain ID	MLST (*if high risk clone)	T3SS effector	Virulence in animal model of bloodstream infection (Allen *et al.* (2020))	Other relevant Characteristics	Reference
P. aeruginosa	PABL004	ST1734	ExoS	medium	Human bacteremia isolate, GenBank assembly: GCA_003411985.2	Scheetz *et al.* (2009)
P. aeruginosa	PABL006	ST235*	ExoU	high	Human bacteremia isolate, GenBank assembly: GCA_003412035.2	Scheetz *et al.* (2009)
P. aeruginosa	PABL012	ST708	ExoS	high	Human bacteremia isolate, GenBank assembly: GCA_003429185.1	Scheetz *et al.* (2009)
P. aeruginosa	PABL048	ST298*	ExoU	medium	Human bacteremia isolate, GenBank assembly: GCA_003411785.2	Scheetz *et al.* (2009)
P. aeruginosa	PABL049	ST244*	ExoS	high	Human bacteremia isolate, GenBank assembly: GCA_003411745.2	Scheetz *et al.* (2009)
P. aeruginosa	PABL054	ST111*	ExoS	medium	Human bacteremia isolate, GenBank assembly: GCA_003411595.2	Scheetz *et al.* (2009)
