# Supplementary material for: Pseudomonas aeruginosa population dynamics in a vancomycin-induced murine model of gastrointestinal carriage
Source: mBio. 2025 Apr 10;16(5):e03136-24. doi: 10.1128/mbio.03136-24 (PMC12077156; doi:10.1128/mbio.03136-24)
Supplement: Table S2 — Primers used in this study. [file mbio.03136-24-s0003.pdf]

Supplemental Table 2. Primers used in this study

Primer name	Primer Sequence 5'-3'
P47	AATGATACGGCGACCACCGAGATCTACACTCTTTCCCTACACGACGCTCTTCCGATCTTGTAAAACGACGGCCAGT
P48	CAAGCAGAAGACGGCATACGAGATCGTGATGTGACTGGAGTTCAGACGTGTGCTCTTCCGATCATTCAGCAGACCTACGATGTCGGGG
P51	CAAGCAGAAGACGGCATACGAGATACATCGGTGACTGGAGTTCAGACGTGTGCTCTTCCGATCATTCAGCAGACCTACGATGTCGGGG
P52	CAAGCAGAAGACGGCATACGAGATGCCTAAGTGACTGGAGTTCAGACGTGTGCTCTTCCGATCATTCAGCAGACCTACGATGTCGGGG
P53	CAAGCAGAAGACGGCATACGAGATTGGTCAGTGACTGGAGTTCAGACGTGTGCTCTTCCGATCATTCAGCAGACCTACGATGTCGGGG
P54	CAAGCAGAAGACGGCATACGAGATCACTGTGTGACTGGAGTTCAGACGTGTGCTCTTCCGATCATTCAGCAGACCTACGATGTCGGGG
P55	CAAGCAGAAGACGGCATACGAGATATTGGCGTGACTGGAGTTCAGACGTGTGCTCTTCCGATCATTCAGCAGACCTACGATGTCGGGG
P56	CAAGCAGAAGACGGCATACGAGATGATCTGGTGACTGGAGTTCAGACGTGTGCTCTTCCGATCATTCAGCAGACCTACGATGTCGGGG
P57	CAAGCAGAAGACGGCATACGAGATTCAAGTGTGACTGGAGTTCAGACGTGTGCTCTTCCGATCATTCAGCAGACCTACGATGTCGGGG
P58	CAAGCAGAAGACGGCATACGAGATCTGATCGTGACTGGAGTTCAGACGTGTGCTCTTCCGATCATTCAGCAGACCTACGATGTCGGGG
P59	CAAGCAGAAGACGGCATACGAGATAAGCTAGTGACTGGAGTTCAGACGTGTGCTCTTCCGATCATTCAGCAGACCTACGATGTCGGGG
P60	CAAGCAGAAGACGGCATACGAGATGTAGCCGTGACTGGAGTTCAGACGTGTGCTCTTCCGATCATTCAGCAGACCTACGATGTCGGGG
P61	CAAGCAGAAGACGGCATACGAGATTACAAGGTGACTGGAGTTCAGACGTGTGCTCTTCCGATCATTCAGCAGACCTACGATGTCGGGG
P62	CAAGCAGAAGACGGCATACGAGATTGTTGACTGTGACTGGAGTTCAGACGTGTGCTCTTCCGATCATTCAGCAGACCTACGATGTCGGGG
P63	CAAGCAGAAGACGGCATACGAGATACGGAACTGTGACTGGAGTTCAGACGTGTGCTCTTCCGATCATTCAGCAGACCTACGATGTCGGGG
P64	CAAGCAGAAGACGGCATACGAGATTCTGACATGTGACTGGAGTTCAGACGTGTGCTCTTCCGATCATTCAGCAGACCTACGATGTCGGGG
P65	CAAGCAGAAGACGGCATACGAGATCGGGACGGGTGACTGGAGTTCAGACGTGTGCTCTTCCGATCATTCAGCAGACCTACGATGTCGGGG
P66	CAAGCAGAAGACGGCATACGAGATGTGCGGACGTGACTGGAGTTCAGACGTGTGCTCTTCCGATCATTCAGCAGACCTACGATGTCGGGG
P67	CAAGCAGAAGACGGCATACGAGATCGTTTCACGTGACTGGAGTTCAGACGTGTGCTCTTCCGATCATTCAGCAGACCTACGATGTCGGGG
P68	CAAGCAGAAGACGGCATACGAGATAAGGCCACGTGACTGGAGTTCAGACGTGTGCTCTTCCGATCATTCAGCAGACCTACGATGTCGGGG
P69	CAAGCAGAAGACGGCATACGAGATTCCGAAACGTGACTGGAGTTCAGACGTGTGCTCTTCCGATCATTCAGCAGACCTACGATGTCGGGG
P70	CAAGCAGAAGACGGCATACGAGATTACGTACGGTGACTGGAGTTCAGACGTGTGCTCTTCCGATCATTCAGCAGACCTACGATGTCGGGG
P71	CAAGCAGAAGACGGCATACGAGATATCCACTCGTGACTGGAGTTCAGACGTGTGCTCTTCCGATCATTCAGCAGACCTACGATGTCGGGG
P72	CAAGCAGAAGACGGCATACGAGATATATCAGTGTGACTGGAGTTCAGACGTGTGCTCTTCCGATCATTCAGCAGACCTACGATGTCGGGG
P73	CAAGCAGAAGACGGCATACGAGATAAAGGAATGTGACTGGAGTTCAGACGTGTGCTCTTCCGATCATTCAGCAGACCTACGATGTCGGGG
	Illumina i7 sequence index Illumina TruSeq Read 2 primer homologous to pminiCTXSTAMP plasmid
P49	ACGCTCTTCCGATCTTGTAAAACGACGGCCAGT
